# Phenomenology, Quantity, and Numerosity

**DOI:** 10.3390/jintelligence11100197

**Published:** 2023-10-10

**Authors:** Marco Bertamini

**Affiliations:** Department of General Psychology, University of Padova, 35131 Padova, Italy; marco.bertamini@unipd.it

**Keywords:** phenomenology, numerosity, perceptual grouping

## Abstract

There are many situations in which we interact with collections of objects, from a crowd of people to a bowl of blackberries. There is an experience of the quantity of these items, although not a precise number, and we have this impression quickly and effortlessly. It can be described as an expressive property of the whole. In the literature, the study of this sense of numerosity has a long history, which is reviewed here with examples. I argue that numerosity is a direct perceptual experience, and that all experiences of numerosity, not only estimations, are affected by perceptual organisation.

## 1. Introduction

According to the Book of Genesis, Jacob asked his father-in-law that all the spotted or speckled sheep would be his wages. Today a spotted breed takes the name of Jacob’s sheep. When he sent a flock to his brother Esau, he instructed his servants to divide them into small groups, so that his gift would appear larger in number: “and said unto his servants, pass over before me, and put a space betwixt drove and drove” (Genesis, 32, 16), cited in Ginsburg (1976). Intuitively, it seems reasonable that dividing a set into many subsets may affect the perceived numerosity. As we shall see, however, the total spacing, rather than the presence of subsets may have been the key factor that produced the desired effect in the Bible story. More importantly, for the story to make sense, we have to assume that Esau trusted his impression of the size of the flock, rather than counting each animal, because if he had counted the sheep the number would have been the same independently of how they were spaced.

The idea of number is associated for most people with mathematics, or with the activity of counting. However, an important question is whether quantity and numerosity are fundamental properties of our perceptual experience, rather than a more cognitive aspect that emerges only in the context of symbolic processing and language.

One way to pose the question is to ask whether we perceive number the same way we perceive other basic properties of the stimuli, spontaneously and effortlessly. Or to use another relevant term, whether these are all emergent properties. For example, when we see a set of dots on a page, we immediately process their size and colour, but the configuration itself also has other perceptual properties; it is seen as random or regular, dense or sparse, and more or less numerous. 

The first part of the paper briefly considers the nature of different qualities, primary, secondary, and tertiary, in relation to numerosity. Next, we outline some of the theories about the “number sense”, in particular the evidence that individuals possess an approximate number system, and then we return to what we can learn about numerosity from a phenomenological perspective.

The first aim of this paper is to provide a historical prospective on the study of numerosity, including some authors whose work in the early part of the 20th century has not yet been translated into English. The second aim is to explore what we can learn about perception of numerosity from a phenomenological perspective. That is, if numerosity is a basic perceptual experience, then it is best placed within the context of perception of shape and size. From this perspective, numerosity is also seen as closely linked to processes of perceptual grouping and size constancy.

A final consideration is about the importance of studying numerosity, and its relevance in everyday life. I started with the example of Jacob, which could be transposed to modern times. From product design to advertising, it is often important to know how people perceive quantity. But perhaps an even more important reason for studying perception of numerosity is its role in development and education. There is evidence that processing of numerosity is not only related to clinical cases of dyscalculia, but it also more generally predicts mathematical abilities (Butterworth et al. 2011; Piazza et al. 2010; Schneider et al. 2017).

## 2. Qualities

In philosophy there is a distinction between primary qualities, independent of the observer, and secondary qualities. The latter qualities produce sensations in the observer, such as colour and taste. These ideas can be traced back to Democritus, and they were developed in Galileo and the British empiricists, such as John Locke (Bianchi and Davies 2019, chps. 3 and 4; Sinico 2015). Locke lists “number” explicitly as an example of primary qualities: “… solidity, extension, motion or rest, number, or figure. These, which I call original or primary qualities of body, are wholly inseparable from it” (Locke 1690). 

Staying in the context of philosophy, tertiary qualities are defined as qualities that exist by virtue of secondary qualities, just as secondary qualities exist by virtue of primary qualities (Blackburn 2008). Another way to talk about tertiary qualities is to stress that the key aspect is their complete subjectivity. A good example of this might be beauty (Alexander 1933). However, a tradition closer to psychology describes tertiary qualities as perceptual aspects of experience, such as the happiness we derive from seeing the sun, and there is a possibility that all observers share these experiences, which means that they are not completely subjective. An even stronger claim about the universality of these qualities comes from the Gestalt tradition (for discussion, see Sinico 2015).

The concept of Gestalt is associated with an early example introduced by von Ehrenfels, the fact that a music melody can be recognized as the same even when all the notes are transposed. Indeed, in Köhler ([1929] 1947) these qualities are called “Ehrenfels qualities”. The concept was further developed by Paolo Bozzi, in particular in relation to naive physics beliefs (for translated text and discussion, see Bianchi and Davies 2019, chp. 11). 

Within Gestalt psychology, therefore, tertiary qualities are expressive, and distant from physical-geometric properties of the stimulus, but can still be treated as fundamental qualities of the phenomenal experience (Bianchi and Davies 2019, chp. 16; Bozzi 1990). Moreover, there is a link between emergent qualities and perceptual organisation, as in the example of the melody. The important aspect of the stimulus is the relationship between the parts, this can be defined in terms of the stimulus, as in the case of the Gestalt grouping principles. However, the process by which separate features are integrated into a whole includes the role of the observer, because there are infinite ways of organising distributed information (over space and time).

After this brief background about terminology, we turn again to the concept of numerosity. As we have seen, one could argue (as Locke did) that number is a primary property as it is independent of the observer, or we could argue that it is a secondary property because the observer integrates separate stimuli into a coherent whole. Indeed, it can be argued that number and quantity are by their essence properties of the whole that do not exist in any of the elements taken separately. Finally, we could argue that certain configurations have a numerosity as part of their expressive qualities, because they can appear scarce or poor, as opposed to rich in number, abundant, etc., and thus numerosity is a tertiary quality. A slightly different reasoning, also leading to the use of the label tertiary quality, is in Prpic and Luccio (2016). They describe numerosity as an immediate impression of power and, therefore, a physiognomic (tertiary) property of the percept.

There are many important observations that show a role of perceptual organisation, and thus of the observer in the perception of numerosity. These observations have been made by many authors over the years and we will discuss in detail some early examples in this paper. In the next section, we briefly describe the more recent developments in the study of numerosity.

## 3. Current Theories

An early view assumed that children learn over time to deal with numbers as part of their cognitive development, as detailed in particular by Piaget (Piaget 1952) in terms of preoperational and operational stages. However, sensitivity to approximate numerosity, or to differences in numerosity, does not require language or the ability to perform operations and it appears to be already present in infancy. This problem led to much interest in the study of this ability, referred to as the approximate number system (ANS) (Burr and Ross 2008; Dehaene 2011). 

Many properties of this system have been studied, and I will list the main findings here. First, we need to distinguish at least three mechanisms that differ from counting and do not rely on symbolic processing. The first one relates to very small sets. There is direct and precise perception of numerosity for small groups (N < 5). For this process the term “subitization” has been created (Kaufman et al. 1949; for early evidence see also Jevons 1871; Liebenberg 1914). The term is a neologism, coined in 1949 from the Latin adjective *subitus* and the Greek suffix *ize* (Kaufman et al. 1949). It captures the immediate sense of knowing how many items are present, and the absence of a process of estimation, and, therefore, the absence of errors. For larger groups of elements (N > 5), we have estimation, and, therefore, the mechanism is called “approximate number sense” (Dehaene 2011). Finally, for high density, the patterns become textures, and individual elements cannot be processed separately (Anobile et al. 2015; Burr et al. 2018).

The approximate number system is present already in infants (Izard et al. 2009; Libertus and Brannon 2010), and similar abilities are shared with many species, including monkeys (Brannon and Terrace 1998), chicks (Rugani et al. 2013), frogs (Stancher et al. 2015), and fish (Piffer et al. 2013). Sensitivity to numerosity is often tested in situations where two sets have to be compared. Performance in this task improves as the difference increases. The ability to discriminate between quantities depends on the ratio of the two quantities, following Weber’s law (Cantlon and Brannon 2006). Studies have also shown that adapting to high numerosity causes underestimation of a subsequent stimulus, while adapting to low numerosity causes overestimation (Burr and Ross 2008).

Brain imaging studies have identified the parietal lobe, and specifically the bilateral intraparietal sulcus, as the region associated with numerical cognition (Dehaene et al. 2003; Prado et al. 2011). Some studies have also shown that activation is largely independent of how numerosity is presented in the stimuli, for instance as circles or triangles (Piazza et al. 2004).

Many aspects of the mechanisms that underpin perception of numerosity are still debated. It is technically challenging to ensure that no other property of the stimuli covary with numerosity, and these sensory correlates may play a role in performance. For example, simply increasing the number of white dots of a configuration will increase total area, total contour length, brightness, etc. A particularly difficult aspect is the influence of density (Anobile et al. 2014; Dakin et al. 2011; Raphael et al. 2013). Another problem is that given a set of elements, it is possible that observers base their responses on sampling (Solomon and Morgan 2018).

This is not the place to review this extensive literature, instead the fundamental observation that we can make is about the direct experience of numerosity. By direct we mean that numerosity is not perceived from counting, from symbolic processing, or on the basis of proxies such as size or brightness. However, it is also clear that many irrelevant dimensions can bias the response to numerosity; these include size of elements (Ginsburg and Nicholls 1988; Shuman and Spelke 2006), regularity of the pattern (Ginsburg 1976), clustering (Bertamini et al. 2018; Guest et al. 2022), topology (He et al. 2015), entropy (DeWind et al. 2020), area (Dakin et al. 2011; Poom et al. 2019; Tokita and Ishiguchi 2010; Vos et al. 1988), and order of presentation (van den Berg et al. 2017). It is still debated whether the interference from irrelevant features arise in early (Balas 2016; Gebuis et al. 2016) or late stages of processing (Harvey and Dumoulin 2017).

## 4. The Phenomenal Experience of Numerosity

In the previous section, we have briefly outlined current research on numerosity. We now take a step back in time. Over a century ago, psychologists were interested in the impression of numerosity, but the focus was on capturing the description of the experience. We will look first at two Italian psychologists whose approach was markedly different: Mario Ponzo (1882–1960) and Silvia De Marchi (1897–1936). Then, we will mention the discovery, 50 years later, of some striking phenomena, described as illusions of numerosity (Solitaire illusion, and Regular-Random Numerosity illusion). We argue that these illusions were discovered thanks to attention to the phenomenology of numerosity.

Mario Ponzo is famous for the illusion that bears his name, one of the best known optical geometrical illusions (Bertamini and Wade 2023; Shapiro and Todorovic 2017; Vicario 2011). Ponzo did not discover the Ponzo illusion. In his writing, he makes clear that the effect was already well known, and he did not claim to have found a new illusion. Instead, he made use of this size contrast effect to study other topics, such as the Moon illusion (Ponzo 1912) and, in more detail, perception of numerosity (Ponzo 1928). 

The 1928 paper, published in German and then in the original Italian in 1929, is titled “Illusioni negli apprezzamenti di collettività” [Illusions in the judgments of numerosity]. I will translate the term “collettività” with numerosity given the recent usage in the literature, alternative translations could be “collection” or “set size”. At the beginning, the author notes that a sense of numerosity is part of many activities in our life. He places numerosity at the same level as time and space, and argues that we have an instinctive tendency to evaluate quantities. It is clear, therefore, that there is a claim about the sense of numerosity as a basic experience that has always been part of our perception (the term used is “primordiale” [“primordial”]).

To have quantity, one has to have separate units, and here Ponzo suggests that these can be created in many ways. In other words, almost anything can take on the value of unity. Next, Ponzo introduces the idea that the best and purest test of perception of numerosity is the direct comparison of two sets, because there is no need at all of counting or even estimating the value of the set of elements. This strategy is indeed common in many studies (e.g., Bertamini et al. 2016; Raphael and Morgan 2016).

**Figure 1:**
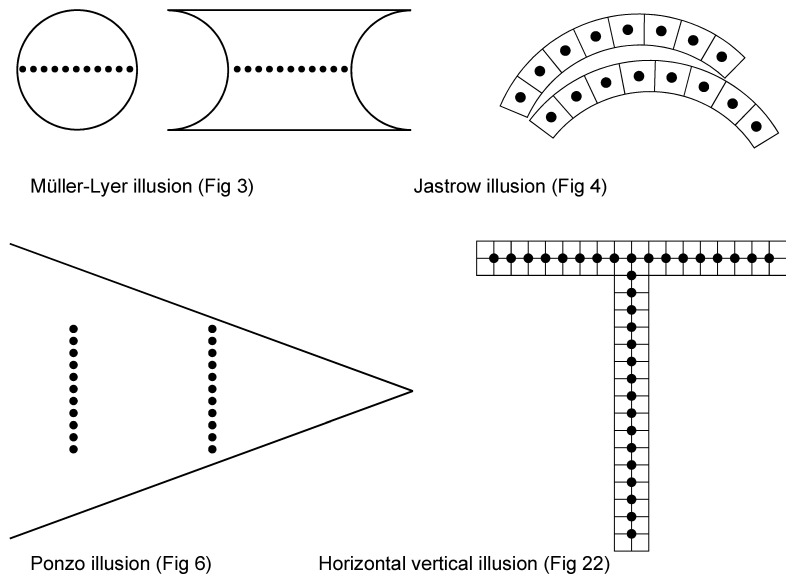
Four examples where the same extent is misperceived as different. In the labels, I have used the names by which these illusions are known today. Figure numbers refer to the images that were adapted from Ponzo (1928) and redrawn by the author (numbers refer to the original). In all cases, when there is a misperception of extent, there is also a misperception of numerosity: more dots are seen for a larger extent.

The paper by Ponzo has 28 figures, and it is the figures that tell the story. Four of them are reproduced in Figure 1. The impression of numerosity is a perception generated by a configuration. Phenomenally, the elements form a whole, and the perceptual organisation process determines many aspects, including the goodness of the whole and its apparent size and shape. It makes sense, therefore, to consider these percepts together when we study the perception of numerosity of the same configuration.

We mentioned in the previous section that judgments of numerosity can be biased by factors such as area, size, and regularity. Instead of seeing these as biases, we can see them as part of the overall impression that we have from objects. Consider again Jacob’s instructions to spread out the sheep so they look more numerous. Assuming this trick worked, was it because of the spreading out over a longer distance in space, a longer interval in time, or was it the creating of subgroups that changed the density and introduced clusters? Let us focus on area, or extent of the group (extent is a better term as the argument applies to both areas and lengths). It is difficult to test the role of extent while controlling for density and inter-element distances. However, what about cases where extent is objectively the same but is perceived as different? If numerosity is fundamentally linked to other properties of the stimuli, and if to assess numerosity we rely, at least in part, on extent, then it follows that any time we misperceive extent, we also misperceive numerosity.

In Figure 1, I have brought together four examples from Ponzo (1928). He used four different effects known already at the time to test the relationship between perception of extent and perception of numerosity: the Müller-Lyer illusion, the Jastrow illusion, the contrast effect now known as the Ponzo illusion, and the horizontal–vertical illusion. One can verify that the perception of numerosity is indeed higher in every case. This is remarkable. 

Let us stay with the Ponzo illusion. Research is still active on this phenomenon (e.g., Cretenoud et al. 2020; Cretenoud et al. 2021; Dobias et al. 2016; Yildiz et al. 2022). There are two main types of explanations, one based on contrast (Fisher 1969; Robinson 1972), and the other based on misapplied size constancy (Gregory 1963). The image used by Ponzo is organised horizontally, because the idea is that we are dealing with a type of contrast effect, and not a misperception of distance. Still, maybe we perceive the elements on the right as closer, a direct prediction from that would be that the individual elements would also appear larger. This does not seem to be the case. The contrast phenomenon that makes the set of elements on the right appear to extend along a greater vertical extent appears to directly affect their numerosity.

In 1929, Ponzo published in the journal *Archivio Italiano Di Psicologia*, the Italian version of his paper on numerosity that had appeared in German in 1928. In the same issue, we find a paper by Silvia De Marchi titled “Le valutazioni numeriche di collettività”. This was translated as “Numerical evaluations of collectivities” by Sergio Masin (2006); but, I will again use the term numerosity here. De Marchi was the first student of Benussi at the University of Padova. In her thesis, we find a careful study of numerosity estimation (Bobbio and Giora 2023; De Marchi 1924). In 1932, Silvia De Marchi married Cesare Musatti, and sadly she died young in 1936. Because of this, and because Benussi had already died in 1927, the pioneering work on numerosity at the University of Padova was interrupted.

What we find in the work of De Marchi is a different approach compared to Ponzo. She is interested in the actual estimation, and the fact that we, at times, have overestimation and underestimation. Because of this, she developed a method of analysing what we now call magnitude estimation data. She discovered that people tend to be consistent, those who underestimate/overestimate do so most of the time. Size of the area and exposure duration matter. Indeed, larger numerosities also tend to produce a subjective shrinking of time in the sense that these stimuli appear to last less. 

Spatial arrangement is important. Here the results are complex, and she notes an interesting interaction between area and density: for small areas, when density decreases, perceived numerosity increases; but, for large areas, a more sparse/less dense stimulus leads to a decrease in perceived numerosity. Finally, De Marchi (1924) observed how knowing in advance the exact number of elements did not change the effect, confirming that people have a direct impression of numerosity, separate and distinct from explicit and propositional knowledge.

It is important but difficult to compare the results from Ponzo and De Marchi. In general, they both describe the nature of a process of estimation or comparison as a natural and fundamental process, and related to perception of shape. One fundamental difference is in the methods, two-alternative forced choice (2AFC) or magnitude estimation. These two methods may tap into different processes, and can lead to different results. This was highlighted by Kanizsa and Luccio (1981) and tested empirically. For example, Agostini and Luccio (1994) found that both perception of length and numerosity in the Müller-Lyer illusion go in the same direction with a forced-choice task, but they go in opposite directions with a magnitude-estimation task.

The idea of different mechanisms has been explored in many studies, for example, on the basis of texturization at higher densities (Anobile et al. 2014), but the systematic work on comparing different tasks has not been followed up. This is, therefore, an area where more work is necessary.

## 5. Two Illusions of Numerosity

**Figure 2:**
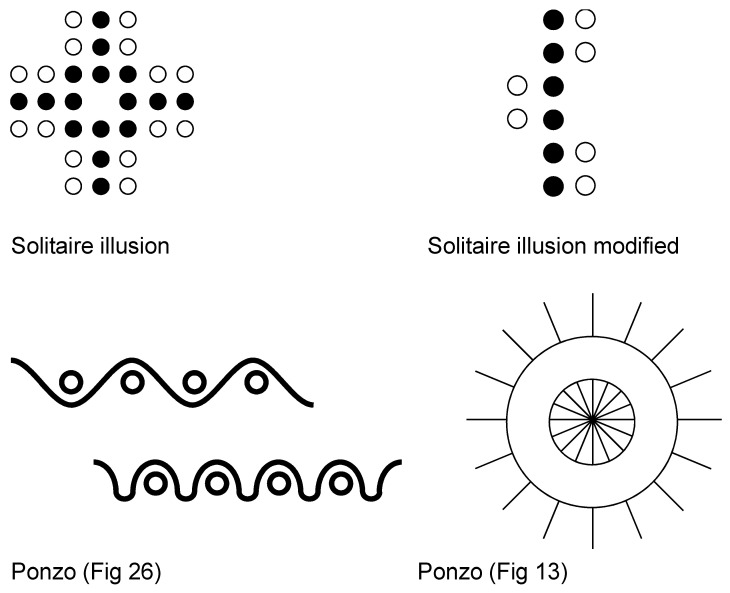
In the Solitaire illusion, we perceive more black dots than white dots. This configuration with 16 elements (top left) is adapted from Frith and Frith (1972). In the original paper, observations were reported with different colours, and also with a version with 12 dots along a line (shown in Figure 3). Kanizsa and Luccio made a version with six dots (adapted here on the top right). On the bottom row, we have two illustrations adapted from Ponzo (1929) and redrawn by the author. The first was also cited and presented in a new version in Kanizsa and Luccio (1981).

The discovery of the Solitaire illusion came about when Chris and Uta Frith received a peg solitaire board game as a Christmas present, with marbles of two different colours. They noticed something about the phenomenal appearance of the marbles when placed in a certain pattern. The configuration is shown in Figure 2 (top left). Note that they were not at the time actively studying perception of numerosity, but they were, as all great scientists, able to make careful observations about the phenomenal appearance of the configuration. Frith and Frith (1972) reported the effect, with supporting empirical data. 

**Figure 3:**
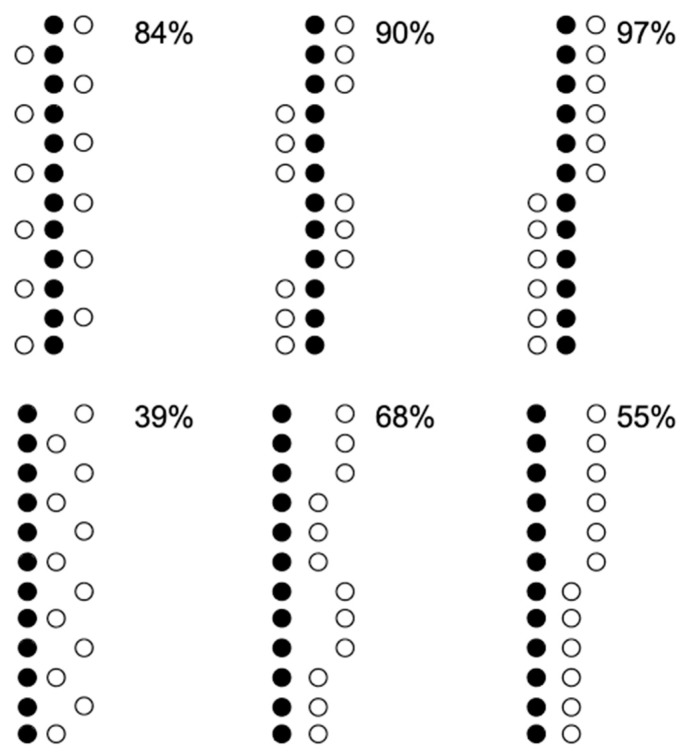
These are variants of the Solitaire illusion adapted from Frith and Frith (1972). They had a sample of 31 observers (18 adults and 13 children). The numbers show the overall percentage of observers who said that the black dots appeared more numerous than the white dots. Note that other colour configurations were also used, and there is nothing special about black in this phenomenon.

The Solitaire illusion is a strong effect, the vast majority of observers see the difference in apparent numerosity, with more elements perceived for the central group. Frith and Frith (1972) argued that the critical factor is that of Gestalt formation. They also created a linear version, with the dots along vertical lines. These are illustrated in Figure 3, together with a summary of the findings. Having elements split into groups with few elements leads to their underestimation, but only if these groups are segmented, that is, if they are placed on the outside of the other group. The effect is reversed in the configuration in the bottom left corner of Figure 3, and this is because here the white dots form a single large group.

In a report from the University of Trieste dated May 1981, Kanizsa and Luccio make use of both the Solitaire illusion and some examples from Ponzo (1929). Unfortunately, this report is not available in English. They argue that the impression of numerosity is not a form of estimation, neither is it secondary to other processes. Instead, it is a primary impression in the same way that figure–ground organisation is primary, and the numerosity is a basic aspect of our phenomenal experience (Kanizsa and Luccio 1981). 

With respect to the examples cited, they make another important observation. We can see these using the stimuli provided in Figure 2. There is a dissociation between the knowledge about the exact number, and the phenomenal impression of numerosity. This is the case because in these versions of the illusions, observers can accurately perceive and report the correct number of elements. Kanizsa and Luccio, in particular, used a redrawing of an image from Ponzo (Figure 26 in the original), and a simplified version of the Solitaire illusion, also shown in my Figure 2. In the first case, we are within the subitizing range (N = 4). In the second case, we have six black and six white circles. Here, also, observers have no problem seeing that to each black element corresponds a white element (a one-to-one match). Given that for these configurations we have no problem reporting the exact number, it is interesting that we can nevertheless have an impression of different numerosity between the two sets. Starting from these observations, Luccio and collaborators conducted some experimental studies in which they found that effects on perceived numerosity that exist for forced-choice comparisons are not replicated with an estimation task (Alam et al. 1986; Agostini and Luccio 1994). 

**Figure 4:**
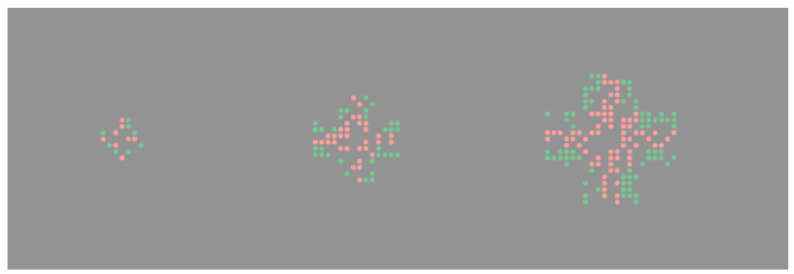
Starting from the original Solitaire illusion configuration, Bertamini et al. (2023) produced versions with higher numerosities (8, 64, 144 per colour). They also tested what happens when the regularity is lost. In these images, half of the dots were removed (therefore, on the left there are only 8 of the original 16 elements per colour, and so on). The illusion was still present for all numerosities, and when only 50% of the dots were shown.

With respect to the Solitaire illusion, the early examples provided by Frith and Frith (1972) included subsets of elements within the subitization range. For example, in the classic configuration (top left of Figure 2) the outside groups have just two elements. However, recently Bertamini et al. (2023) have shown that the effect is very robust and extends to cases with much higher numerosities. It also remains strong when the arrangement is not regular, as shown in Figure 4.

In Figure 2, I have included one more example from Ponzo (1928). The image shows a circle with radii extending from the centre (bottom right). Ponzo observed that we have an impression that there are more radii in the middle than on the outside. We can, therefore, repeat the point made earlier, about the fact that the impression remains despite our awareness that the numerosity is the same in both cases. But here we can make an additional point. There are also perceptual factors that specify a one-to-one match between the lines inside and outside the ring, because of good continuation. Indeed, the ring can be perceived as an occluder, and the lines seen outside are just the phenomenal continuation of the lines inside. Despite this perceptual unity of the elements, the impression of a difference in numerosity remains. This is a challenge for all theories of numerosity. However, I should note that the extent by which perception of numerosity changes when the lines are seen to continue under an occluder has not been experimentally tested yet.

In considering the work by De Marchi (1924, 1929), Kanizsa and Luccio (1981), and the implications of some of Ponzo (1928) examples, we have seen an interesting dissociation between precision in numerosity estimation and phenomenal sense of numerosity. These observations have been forgotten in the more recent literature, and current models of numerosity do not provide an explanation for this dissociation. As already noted in the previous section, more theoretical and experimental work is necessary.

**Figure 5:**
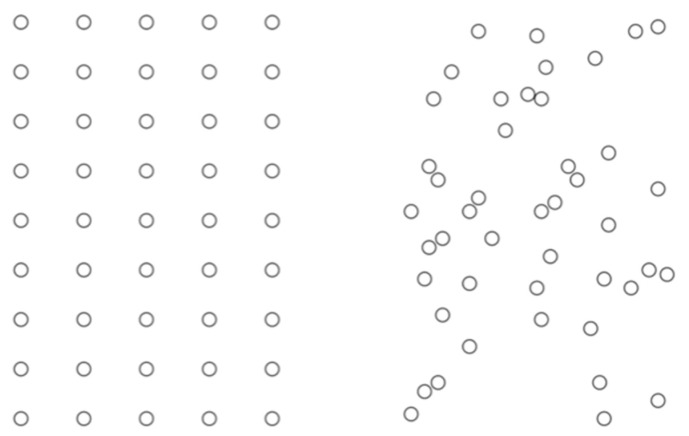
Most observers report that the configuration on the left appears more numerous than the one on the right. This example is adapted from Ginsburg (1980).

In addition to the Solitaire illusion, there is another numerosity illusion reported approximately in the same period. The regular-random numerosity illusion is the tendency for regularly arranged patterns to appear more numerous than randomly arranged patterns (Ginsburg 1976, 1980, 1991). A redrawing is shown in Figure 5. Although in the original demonstrations the dots formed a very regular and symmetrical pattern, later studies have extended the result, confirming that the key factor is not the regularity but the clustering (Bertamini et al. 2016; Chakravarthi and Bertamini 2020; Valsecchi et al. 2013). Indeed, other things being equal, symmetric dot patterns appear less numerous than random dot patterns (Apthorp and Bell 2015).

Let us return to Jacob and the Bible. We anticipated the difficulty of knowing what kind of grouping effect leads to a bias in perception of numerosity. The Solitaire illusion shows that the set with small groups appears less numerous than the single central group, similar grouping effects are present in Ponzo. And for larger and less regular sets, the regularity illusion again shows that making small clusters of elements makes the stimulus appear less numerous than a stimulus in which elements are spaced out. Local clustering was studied, for example, in Bertamini et al. (2016), and grouping by distance, colour, or motion by Poom et al. (2019).

Could the Bible be wrong? It is possible to make configurations appear more numerous by increasing the distance between elements? We saw this effect of overall extent in De Marchi and in many other studies. Ponzo demonstrated the role of extent elegantly by manipulating subjective extent. Therefore, Jacob may have achieved his goal because of the increased overall space placed between the sheep, not because of grouping.

## 6. Conclusions

In the introduction, we asked the question of whether quantity and numerosity are fundamental properties of our perceptual experience, rather than a cognitive aspect that emerges only thanks to symbolic processing. We have seen that the literature has identified at least three kinds of perception of numerosity: subitization, estimation, and texturisation. These are all fast processes and are distinct from counting, which would be a fourth kind. 

The study of perception of numerosity is important for many reasons. Indeed, the thesis that De Marchi wrote in 1924 had the title “Contributi alla psicologia giudiziaria” [Contributions to forensic psychology]. This reflects the complex and rich nature of the thesis. It includes a discussion of why it is important to study errors that people make on simple judgments. When estimating numerosity, people can be confident and reliable, and yet show systematic biases. De Marchi also noted that if we divide people into two groups, those who tend to underestimate and those who tend to overestimate, these tendencies are stable over time. 

In the more recent literature, there are many carefully designed experiments. The approach based on the psychophysics toolbox has been fruitful. For example, it has been shown that Weber’s law applies only within a certain range of density values. On this evidence, we can argue for separate mechanisms (Anobile et al. 2014). Another very important approach comes from neuroscience. Imagining studies have identified brain regions probably specialised for perception of numerosity (Piazza et al. 2004). However, it is important not to forget the careful description of the perceptual experience. It is from this analysis that we first realised that numerosity is perceived immediately and directly.

We have seen that the interest in the study of perception of quantity and numerosity has a long history. Some early accounts have not received enough attention, in part because they were not available in English (this was the case for Ponzo’s paper, only recently translated: Bertamini and Wade 2023; and for De Marchi, whose work is still largely unavailable in translation). For visual stimuli where the elements are clearly segmented (i.e., not for textures), there is strong evidence that people have an impression of numerosity that is fast and spontaneous, not based on other dimensions such as size or brightness; although, these other dimensions can produce biases. We have also seen that some strong effects, described as illusions, provide evidence of the expressive nature of configurations, linked to perceptual grouping. 

It is interesting to contrast two views. In the mainstream literature on numerosity, the biases due to various cues are seen as problematic. They are problematic because a direct sense of number needs to be abstract from the accidental features of the stimuli (the shape of the elements) and of the configuration (its overall area or its density). That many biases exist is beyond dispute, and we have seen in Section 3 that the debate is whether these forms of “interference” happen early or late during the process of perception of numerosity. According to this view, therefore, unbiased perception of numerosity is seen as normative, and any deviation is seen as the product of other mechanisms that are not specifically about numerosity. Moreover, the biases are seen as affecting the estimation process, in the case of uncertainty. But as we have seen, the experience of numerosity can vary even in cases where there is no uncertainty. An alternative view, much older historically, is that these aspects of perception of numerosity are not “problems” and that these effects are not a form of “interference”. Instead, numerosity in the phenomenal sense is an experience intrinsically tied to the whole, or to use the traditional term, to the Gestalt. 

## Data Availability

Not applicable.
